# Disproportionate adverse event signals of selumetinib in neurofibromatosis type I: insights from FAERS

**DOI:** 10.3389/fphar.2024.1454418

**Published:** 2025-01-07

**Authors:** Lin Li

**Affiliations:** Department of Oncology, Zibo Municipal Hospital, Zibo, ShanDong, China

**Keywords:** selumetinib, neurofibromatosis type I, FAERS, adverse reactions, rare diseases

## Abstract

**Background:**

Neurofibromatosis type 1 (NF1) is a rare neurogenetic disorder with limited treatment options. Selumetinib, a MEK1/2 inhibitor, has emerged as a promising therapy for inoperable NF1-related plexiform neurofibromas.

**Methods:**

Our retrospective pharmacovigilance study utilized the FDA Adverse Event Reporting System (FAERS) to comprehensively evaluate Selumetinib’s safety profile in real-world settings. Data from the third quarter of 2020 to the fourth quarter of 2023 were analyzed, identifying 498 adverse event reports with Selumetinib as the primary suspect drug.

**Results:**

Statistical analysis revealed disproportionate signals for skin and subcutaneous tissue disorders, eye disorders, and various congenital, familial, and genetic disorders. The most common adverse events were elevated blood creatine phosphokinase, rash, and acneiform dermatitis. Notably, several adverse events, including rhabdomyolysis, were identified but not listed on the Selumetinib product label, based on a comparison with the FDA drug labeling.

**Conclusion:**

The study underscores the importance of early detection and management of adverse reactions associated with Selumetinib, particularly within the initial month of treatment. These findings provide valuable insights for clinicians and regulators to ensure the safe and effective use of Selumetinib in NF1 patients.

## Introduction

Neurofibromatosis type 1 (NF1), or von Recklinghausen disease, is a neurogenetic disorder caused by mutations in the NF1 gene, inherited in an autosomal dominant manner (Ibrahim et al., 2024; Cichowski et al., 2003). It has a global incidence of approximately 1 in 3,000 individuals, with about 50% of cases resulting from familial mutations. NF1 typically presents with café-au-lait macules, multiple neurofibromas, and axillary or inguinal freckling, with neurofibromas being the most common symptom (Legius et al., 2021). Approximately 20%–50% of patients develop plexiform neurofibromas (PNs), which can cause pain, neurological dysfunction, skeletal deformities, and disfigurement (Colombo et al., 2022).

Although surgery remains the primary treatment, complete surgical resection is challenging due to the unclear boundaries between PN and adjacent tissues. Moreover, the effectiveness of conventional radiotherapy and chemotherapy is limited in treating NF1 (Janinis et al., 2003; Vasudevan et al., 2024), and partial types of chemotherapy and radiotherapy can even induce secondary tumors in these patients, further complicating disease management. For patients who are suitable for surgery, the tumor’s location, size, and invasiveness often preclude complete resection. As a result, partial resection frequently leads to a recurrence rate of more than 50%. The median progression-free survival (PFS) for partially resected PN is less than 1.5 years, with only 10%–40% of patients achieving a 2-year PFS. Consequently, treating inoperable and residual PN remains a significant clinical challenge. However, recent research on biological targets and clinical trials has opened new avenues for diagnosis and treatment.

NF1 gene mutations lead to the inactivation or downregulation of neurofibromin, a negative regulator of the RAS system (Downward, 2003; Dasgupta et al., 2005), which results in excessive activation of the RAS system and tumor development. MEK inhibitors can reduce the activity of the RAF-MEK-ERK pathway, thereby inhibiting tumor growth. Selumetinib, a MEK1/2 inhibitor, targets the downstream effector protein MEK in the RAS-RAF-MEK-ERK (MAPK) pathway and can be used against various tumors with mutations in this pathway (Church et al., 2024; Ram et al., 2023). A phase II trial on Selumetinib for inoperable NF1-related plexiform neurofibromas (PNs) was published in The New England Journal of Medicine in 2020. This open-label study included 50 patients aged 2–18 years (Gross et al., 2023). After administering Selumetinib at 25 mg/m^2^ every 12 h in 28-day cycles, 70% achieved a partial response, and 56% had a durable response. The three-year progression-free survival rate was 84%. Due to its efficacy and safety, Selumetinib received breakthrough therapy designation and orphan drug status from the FDA, becoming the first approved treatment for symptomatic, inoperable NF1-related PNs in patients aged 2–18 (Gross et al., 2023; Guo et al., 2024; Gross et al., 2022).

While Selumetinib has achieved significant success in clinical applications, its side effects have increasingly garnered attention (Kassotis et al., 2023). Common adverse events, such as gastrointestinal disturbances, skin reactions, and elevated creatine phosphokinase levels, require careful monitoring. Serious complications, including heart failure and ocular toxicity, though less common, also warrant vigilance. However, due to the low incidence of NF1, the limited study population, and varying approval statuses across different regions, the sample size in real-world studies remains small (Wang et al., 2024; Suenobu et al., 2023). Therefore, further clarification of the adverse events associated with Selumetinib is particularly important.

To ensure the safety of clinical drug use and mitigate adverse reactions in patients, this study examined the adverse reactions of Selumetinib using the US Food and Drug Administration (FDA) Adverse Event Reporting System (FAERS) database (Dhodapkar et al., 2022). By employing disproportionality analysis methods, we seek to identify potential safety signals, explore plausible mechanisms underlying serious adverse events, and provide actionable insights for clinical management. The findings are expected to fill critical gaps in post-market pharmacovigilance research and contribute to improving patient safety.

## Materials and methods

### Data sources and procedures

**FIGURE 1:**
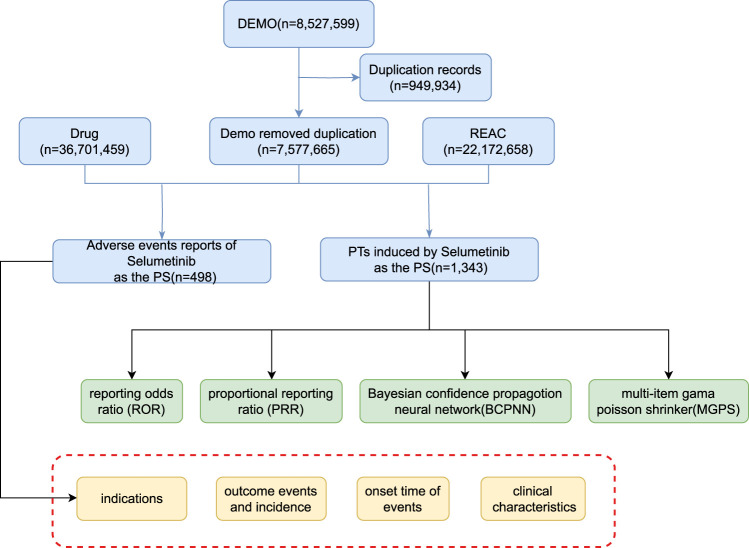
The flow diagram of selumetinib-related AEs from FAERS database.

We conducted a retrospective pharmacovigilance study using FAERS quarterly data from the third quarter of 2020 (July 2020) to the fourth quarter of 2023 (December 2023). Participant selection criteria are outlined in Figure 1. Cases were identified using a combination of generic and proprietary drug names. Search terms for Selumetinib included Selumetinib, SELUMETINIB, and Selumetinib bisulfate to retrieve adverse event (AE) reports. Due to the database’s reliance on voluntary reporting, duplicate reports were possible. Our study processed the original data to remove duplicates (n = 949,934), resulting in a dataset of reports where Selumetinib was the primary suspect (PS) drug. This study adhered to the principles of the Adverse events were coded using the Medical Dictionary for Regulatory Activities (MedDRA) Preferred Terms (PT; version 26.1) at the 15-level. Only drugs reported as “primary suspect products” were included in the analysis. AEs and AE categories were defined by PT and “System Organ Class (SOC),” respectively. The AE occurrence date and the start date of Selumetinib treatment were used to determine the onset time. The relevant statistical analysis was performed using “faersR”.

### Statistical analysis and signals detection

In this study, signal mining of drug-induced adverse events was conducted using the proportional imbalance measurement method. Techniques such as the reported odds ratio (ROR), proportional reported odds ratio (PRR), and Bayesian confidence propagation neural network (BCPNN) were employed (Bate and Evans, 2009; Küçükosmanoglu et al., 2024; Evans et al., 2001; Wilson et al., 2004). This study employed disproportionality analysis methods to identify potential adverse event (AE) signals associated with Selumetinib. The following signal detection methods were used: ROR measures the odds of reporting a specific AE for Selumetinib compared to other drugs in the database. A signal is considered significant when the lower limit of the 95% confidence interval (CI) of the ROR exceeds 1. PRR compares the proportion of a specific AE associated with Selumetinib to its proportion with other drugs. A signal is deemed significant if PRR ≥2, the AE count ≥3, and the χ^2^ statistic ≥4. BCPNN calculates the Information Component (IC) to assess the disproportionality of AEs. A signal is considered positive when the lower limit of the 95% credibility interval of IC (IC025) > 0. Empirical Bayes Geometric Mean (EBGM): EBGM uses Bayesian shrinkage to stabilize disproportionality estimates for small sample sizes. A signal is positive when the EBGM lower limit (EB05) exceeds 1. Thresholds and Rationale: The thresholds for these methods were chosen based on their widespread validation in pharmacovigilance research. ROR and PRR provide intuitive measures of signal strength, while BCPNN and EBGM account for variability in the data, enhancing robustness (Supplementary Table S1). Considering a low number of expected cases could lead to an insufficient sensitivity to detect disproportionality of relevant strength, we reported the sensitivity of a representative IC025 to measure the reliability of results. The result of PT was deemed confident when the sensitivity to detect representative IC025 > 0.8 (Trillenberg et al., 2023).

## Results

### Overview

**FIGURE 2:**
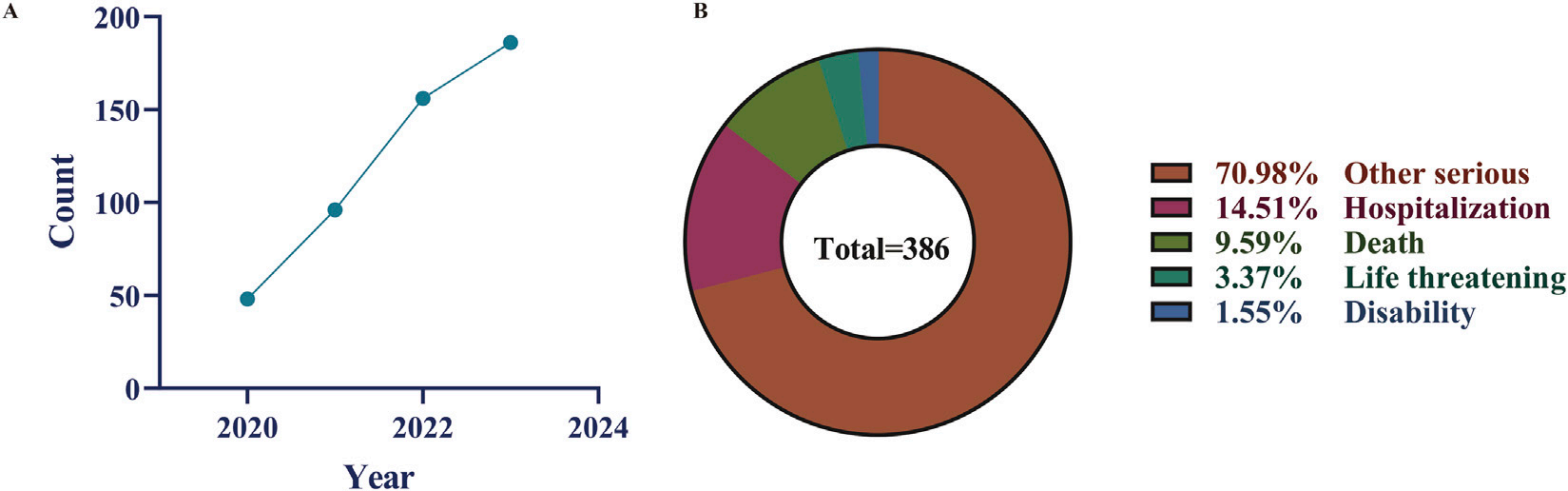
Some clinical characteristics of selumetinib reported in the FAERS database. **(A)** time-series plot was introduced to illustrate the trend of AE reports over the study period (2020–2023); **(B)** Distribution of selumetinib-related adverse event outcomes.

From the third quarter of 2020 to the fourth quarter of 2023, our study obtained a total of 8,527,599 adverse event reports from the FAERS database. After removing 949,934 duplicate reports, 498 reports out of the remaining 7,577,665 cases listed Selumetinib as the “primary suspect” drug (Figure 1). Table 1 provides an overview of the reported adverse events associated with Selumetinib. The reported adverse events occurred in 2020 (n = 48, 9.88%), 2021 (n = 96, 19.75%), 2022 (n = 156, 32.10%), and 2023 (n = 186, 38.27%) (Figure 2A). Males (42.59%) accounted for a higher proportion of adverse events compared to females. The highest number of reported adverse events came from the United States (64.81%). Serious outcomes included hospitalization, death, life-threatening conditions, disability, and other severe outcomes. Hospitalization (14.51%) was the most frequently reported serious outcome, followed by death (9.59%) (Figure 2B). Adverse events were predominantly reported by physicians, consumers, and healthcare professionals (45.88%, 33.13%, and 18.93%, respectively).

**TABLE 1:** Summary of clinical features of the cases.

Variable	Total
Sex	
Female	180 (37.04)
Male	207 (42.59)
Unknown	99 (20.37)
Age(Year)	
<=18	192 (39.51)
>18,<=60	50 (10.29)
>60	9 (1.85)
Unknow	235 (48.35)
Reporter	
Physician	223 (45.88)
Consumer	161 (33.13)
Pharmacist	92 (18.93)
Unknown	10 (2.06)
Region of AE	
United States	315 (64.81)
Other	171 (35.19)
Outcomes	
Other serious	274 (70.98)
Hospitalization	56 (14.51)
Death	37 (9.59)
Life threatening	13 (3.37)
Disability	6 (1.55)
Weight	51.95 (34.35,64.93)

### Signal detection

#### Analysis of adverse event signals involving SOC

A total of 15 SOC categories were involved in the adverse event signals identified in this study (Table 2). The categories with the highest number of signals and reports included skin and subcutaneous tissue disorders, general disorders and administration site conditions, and gastrointestinal disorders. These findings are consistent with the descriptions in the Selumetinib package insert, indicating the feasibility of our research methods and the reliability of our results. Among these, skin and subcutaneous tissue disorders had the highest number of signals and reports, significantly surpassing other SOCs, suggesting that these adverse reactions are the most common in clinical practice. This underscores the need for heightened attention to such adverse reactions during patient treatment.

**TABLE 2:** The signal strength of Selumetinib at the system organ class (SOC) level.

SOC	Case	ROR (95% CI)	PRR (95% CI)	IC(IC025)	EBGM(EBGM05)
Skin And Subcutaneous Tissue Disorders	223	3.72 (3.22, 4.29)	3.26 (2.9, 3.67)	1.7 (1.5)	3.26 (2.89)
General Disorders And Administration Site Conditions	173	0.67 (0.57, 0.78)	0.71 (0.62, 0.81)	−0.49 (-0.72)	0.71 (0.62)
Gastrointestinal Disorders	144	1.41 (1.19, 1.68)	1.37 (1.17, 1.6)	0.45 (0.2)	1.37 (1.18)
Investigations	139	1.81 (1.52, 2.16)	1.73 (1.48, 2.02)	0.79 (0.54)	1.72 (1.49)
Infections And Infestations	88	1.16 (0.93, 1.44)	1.15 (0.95, 1.4)	0.2 (-0.11)	1.15 (0.96)
Injury, Poisoning And Procedural Complications	84	0.46 (0.37, 0.57)	0.49 (0.39, 0.61)	−1.02 (-1.34)	0.49 (0.41)
Nervous System Disorders	72	0.72 (0.57, 0.91)	0.73 (0.59, 0.91)	−0.45 (-0.79)	0.73 (0.6)
Neoplasms Benign, Malignant And Unspecified	70	1.12 (0.88, 1.42)	1.11 (0.88, 1.4)	0.15 (-0.19)	1.11 (0.91)
Eye Disorders	54	2.11 (1.61, 2.77)	2.06 (1.6, 2.66)	1.05 (0.66)	2.06 (1.64)
Musculoskeletal And Connective Tissue Disorders	48	0.66 (0.5, 0.89)	0.68 (0.52, 0.89)	−0.56 (-0.97)	0.68 (0.53)
Cardiac Disorders	36	1.37 (0.98, 1.91)	1.36 (0.99, 1.86)	0.44 (-0.03)	1.36 (1.03)
Psychiatric Disorders	31	0.39 (0.27, 0.55)	0.4 (0.28, 0.57)	−1.32 (-1.83)	0.4 (0.3)
Metabolism And Nutrition Disorders	30	1.17 (0.82, 1.69)	1.17 (0.82, 1.66)	0.23 (-0.29)	1.17 (0.86)
Respiratory, Thoracic And Mediastinal Disorders	28	0.45 (0.31, 0.65)	0.46 (0.32, 0.67)	−1.12 (-1.65)	0.46 (0.34)
Renal And Urinary Disorders	26	1.03 (0.7, 1.52)	1.03 (0.71, 1.49)	0.05 (-0.5)	1.03 (0.75)
Blood And Lymphatic System Disorders	22	0.94 (0.62, 1.43)	0.94 (0.62, 1.42)	−0.09 (-0.69)	0.94 (0.66)
Vascular Disorders	15	0.59 (0.35, 0.98)	0.59 (0.35, 0.98)	−0.75 (-1.46)	0.59 (0.39)
Reproductive System And Breast Disorders	11	1.4 (0.78, 2.54)	1.4 (0.78, 2.52)	0.49 (-0.33)	1.4 (0.85)
Hepatobiliary Disorders	8	0.73 (0.36, 1.46)	0.73 (0.37, 1.45)	−0.45 (-1.4)	0.73 (0.41)
Congenital, Familial And Genetic Disorders	8	2.21 (1.1, 4.44)	2.21 (1.11, 4.39)	1.14 (0.2)	2.21 (1.23)
Ear And Labyrinth Disorders	4	0.72 (0.27, 1.93)	0.72 (0.27, 1.92)	−0.47 (-1.73)	0.72 (0.32)

#### Analysis of adverse event signals involving PT

**FIGURE 3:**
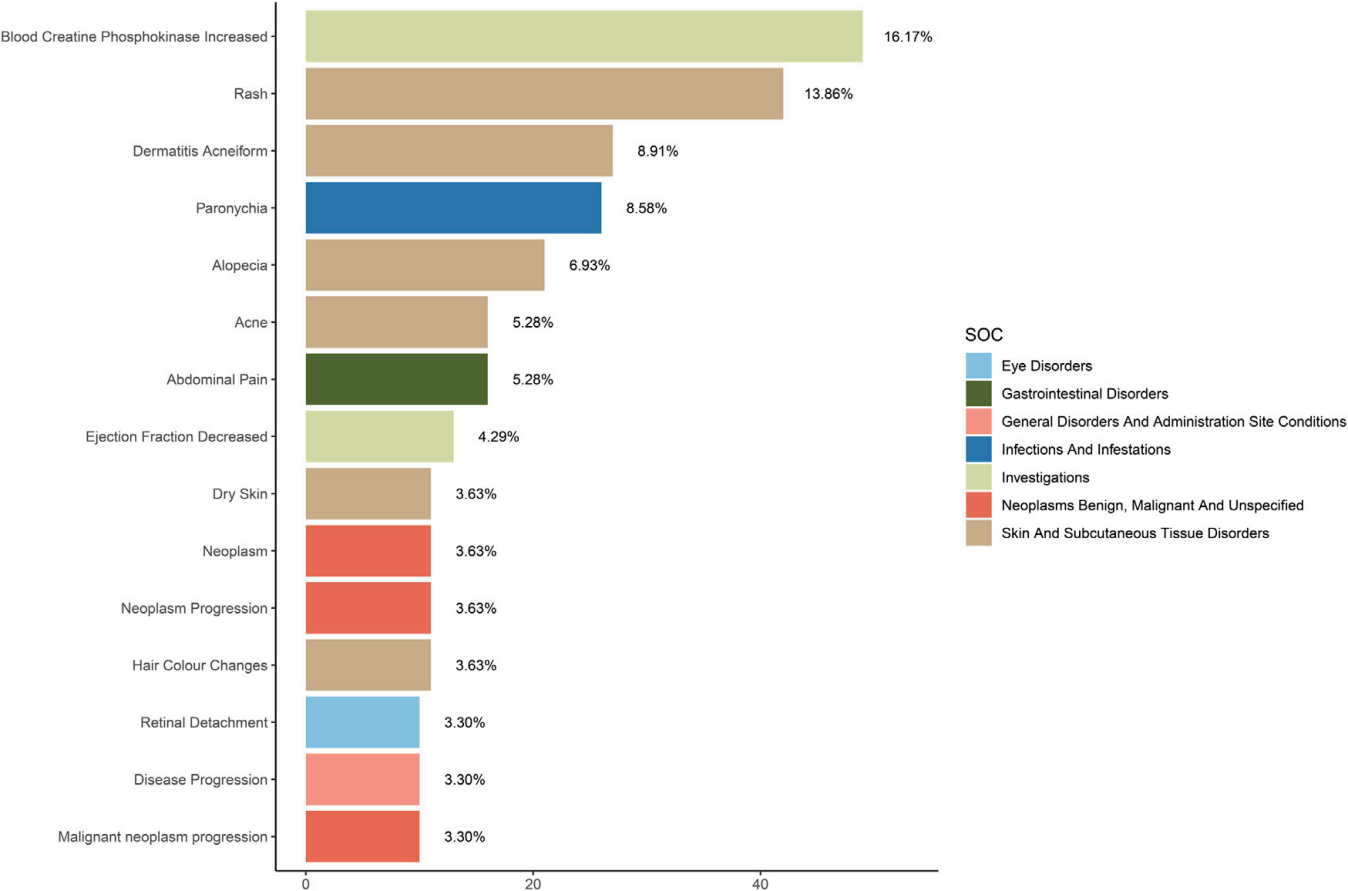
The top 15 SOCs ranked by report numbers.

In our statistical results, the most common AEs were elevated blood creatine phosphokinase (11.40%, n = 49), rash (9.76%, n = 42), acneiform dermatitis (6.27%, n = 27), paronychia (6.04%, n = 26), alopecia (4.88%, n = 21), abdominal pain (3.72%, n = 16), and deep vein thrombosis (3.72%, n = 16) (Figure 3). Among the 48 positive PTs in this study, three AEs had case counts were not listed on the Selumetinib product label, including peripheral edema (n = 8), rhabdomyolysis (n = 6), and frequent urination (n = 5) (Table 3). Further sensitivity analysis showed that all PTs passed the sensitivity analysis.

**TABLE 3:** The PTs ranked by report numbers.

SOC	PT	Case	ROR (95% CI)	PRR (95% CI)	IC(IC025)	EBGM(EBGM05)
Investigations	Blood Creatine Phosphokinase Increased	49	124.92 (93.78, 166.41)	120.32 (91.45, 158.31)	6.9 (6.49)	119.13 (93.72)
Skin And Subcutaneous Tissue Disorders	Rash	42	4.62 (3.39, 6.28)	4.5 (3.35, 6.04)	2.17 (1.73)	4.5 (3.48)
Skin And Subcutaneous Tissue Disorders	Dermatitis Acneiform	27	226.71 (154.33, 333.03)	222.08 (153.03, 322.29)	7.77 (7.22)	218.06 (158.06)
Infections And Infestations	Paronychia	26	282.89 (191.03, 418.92)	277.33 (187.39, 410.43)	8.08 (7.53)	271.07 (195.17)
Skin And Subcutaneous Tissue Disorders	Alopecia	21	5.26 (3.42, 8.1)	5.2 (3.38, 8)	2.38 (1.77)	5.19 (3.62)
Skin And Subcutaneous Tissue Disorders	Acne	16	11.59 (7.08, 18.99)	11.47 (7.03, 18.72)	3.52 (2.83)	11.46 (7.58)
Gastrointestinal Disorders	Abdominal Pain	16	3.55 (2.17, 5.81)	3.52 (2.16, 5.75)	1.81 (1.12)	3.52 (2.33)
Investigations	Ejection Fraction Decreased	13	33.4 (19.33, 57.72)	33.08 (19.11, 57.27)	5.04 (4.28)	32.99 (20.88)
Skin And Subcutaneous Tissue Disorders	Dry Skin	11	4.34 (2.4, 7.85)	4.31 (2.39, 7.76)	2.11 (1.29)	4.31 (2.62)
Neoplasms Benign, Malignant And Unspecified	Neoplasm	11	39.36 (21.72, 71.32)	39.04 (21.68, 70.29)	5.28 (4.46)	38.92 (23.67)
Neoplasms Benign, Malignant And Unspecified	Neoplasm Progression	11	9.42 (5.2, 17.06)	9.35 (5.19, 16.83)	3.22 (2.4)	9.35 (5.69)
Skin And Subcutaneous Tissue Disorders	Hair Colour Changes	11	44.63 (24.63, 80.88)	44.27 (24.59, 79.7)	5.46 (4.64)	44.11 (26.82)
Eye Disorders	Retinal Detachment	10	58 (31.09, 108.21)	57.57 (30.75, 107.79)	5.84 (4.98)	57.29 (34)
General Disorders And Administration Site Conditions	Disease Progression	10	3.44 (1.85, 6.42)	3.43 (1.83, 6.42)	1.78 (0.92)	3.42 (2.03)
Neoplasms Benign, Malignant And Unspecified	Malignant Neoplasm Progression	10	4.14 (2.22, 7.71)	4.12 (2.2, 7.71)	2.04 (1.18)	4.12 (2.45)
Investigations	Aspartate Aminotransferase Increased	8	9.23 (4.61, 18.5)	9.18 (4.62, 18.23)	3.2 (2.25)	9.18 (5.13)
Skin And Subcutaneous Tissue Disorders	Eczema	8	6.89 (3.44, 13.81)	6.85 (3.45, 13.6)	2.78 (1.83)	6.85 (3.83)
General Disorders And Administration Site Conditions	Oedema Peripheral	8	4.78 (2.39, 9.59)	4.76 (2.4, 9.45)	2.25 (1.3)	4.76 (2.66)
Neoplasms Benign, Malignant And Unspecified	Neurofibrosarcoma	8	2534.93 (1180.36, 5,444)	2519.55 (1173.14, 5411.25)	11.02 (9.99)	2081.54 (1098.07)
Gastrointestinal Disorders	Stomatitis	7	5.08 (2.42, 10.67)	5.06 (2.4, 10.66)	2.34 (1.33)	5.05 (2.71)
Musculoskeletal And Connective Tissue Disorders	Rhabdomyolysis	6	9.18 (4.11, 20.47)	9.14 (4.09, 20.41)	3.19 (2.12)	9.13 (4.67)
Congenital, Familial And Genetic Disorders	Neurofibromatosis	6	1849.62 (781.74, 4376.29)	1841.21 (777.26, 4361.53)	10.64 (9.49)	1595.85 (776.31)
Neoplasms Benign, Malignant And Unspecified	Neurofibroma	6	1759.4 (745.68, 4151.19)	1751.39 (739.35, 4148.76)	10.58 (9.43)	1527.94 (745.01)
Skin And Subcutaneous Tissue Disorders	Skin Disorder	6	7.31 (3.28, 16.31)	7.28 (3.26, 16.26)	2.86 (1.79)	7.28 (3.72)
Renal And Urinary Disorders	Pollakiuria	5	6.45 (2.68, 15.51)	6.42 (2.66, 15.51)	2.68 (1.53)	6.42 (3.08)
Skin And Subcutaneous Tissue Disorders	Dermatitis	4	9.81 (3.67, 26.19)	9.78 (3.67, 26.06)	3.29 (2.02)	9.78 (4.3)
Cardiac Disorders	Pericardial Effusion	4	8.79 (3.29, 23.45)	8.76 (3.29, 23.34)	3.13 (1.86)	8.76 (3.85)
Neoplasms Benign, Malignant And Unspecified	Tumour Pain	4	121.87 (45.44, 326.81)	121.5 (45.6, 323.73)	6.91 (5.64)	120.29 (52.69)
Metabolism And Nutrition Disorders	Hyperphosphataemia	4	84.98 (31.74, 227.56)	84.73 (31.8, 225.76)	6.39 (5.12)	84.14 (36.9)
Skin And Subcutaneous Tissue Disorders	Ingrowing Nail	4	75.5 (28.21, 202.07)	75.27 (28.25, 200.55)	6.23 (4.95)	74.81 (32.82)
Eye Disorders	Serous Retinopathy	4	889.2 (321.58, 2458.76)	886.51 (319.93, 2456.5)	9.69 (8.37)	825.44 (352.44)
Skin And Subcutaneous Tissue Disorders	Skin Toxicity	4	30.35 (11.36, 81.08)	30.26 (11.36, 80.63)	4.92 (3.65)	30.19 (13.26)
Cardiac Disorders	Cardiotoxicity	4	17.27 (6.47, 46.11)	17.22 (6.46, 45.88)	4.1 (2.84)	17.2 (7.56)
General Disorders And Administration Site Conditions	Impaired Healing	4	6.19 (2.32, 16.53)	6.18 (2.32, 16.47)	2.63 (1.36)	6.17 (2.72)
Skin And Subcutaneous Tissue Disorders	Rash Maculo-Papular	3	6.7 (2.16, 20.81)	6.69 (2.15, 20.85)	2.74 (1.32)	6.69 (2.59)
Psychiatric Disorders	Personality Change	3	19.45 (6.26, 60.44)	19.41 (6.23, 60.5)	4.28 (2.86)	19.38 (7.5)
Investigations	Blood Bilirubin Increased	3	7.2 (2.32, 22.36)	7.19 (2.31, 22.41)	2.84 (1.43)	7.18 (2.78)
Reproductive System And Breast Disorders	Menstrual Disorder	3	22.63 (7.28, 70.33)	22.58 (7.24, 70.38)	4.49 (3.08)	22.54 (8.73)
Nervous System Disorders	Hydrocephalus	3	31.84 (10.24, 99.01)	31.77 (10.19, 99.02)	4.99 (3.57)	31.69 (12.27)
Infections And Infestations	Pharyngitis Streptococcal	3	13.56 (4.36, 42.12)	13.53 (4.34, 42.17)	3.76 (2.34)	13.51 (5.23)
Nervous System Disorders	Intracranial Pressure Increased	3	27.98 (9, 86.98)	27.92 (8.96, 87.02)	4.8 (3.38)	27.86 (10.78)
Infections And Infestations	Skin Infection	3	11.09 (3.57, 34.45)	11.07 (3.55, 34.5)	3.47 (2.05)	11.06 (4.28)
Infections And Infestations	Rash Pustular	3	36.09 (11.61, 112.24)	36.01 (11.55, 112.24)	5.17 (3.75)	35.91 (13.9)
Gastrointestinal Disorders	Mouth Ulceration	3	6.82 (2.2, 21.17)	6.8 (2.18, 21.19)	2.77 (1.35)	6.8 (2.63)
Renal And Urinary Disorders	Proteinuria	3	7.05 (2.27, 21.9)	7.04 (2.26, 21.94)	2.81 (1.4)	7.03 (2.73)
Neoplasms Benign, Malignant And Unspecified	Neoplasm Recurrence	3	43.51 (13.99, 135.36)	43.41 (13.93, 135.3)	5.43 (4.02)	43.26 (16.74)
General Disorders And Administration Site Conditions	Mucosal Inflammation	3	5.95 (1.92, 18.49)	5.94 (1.91, 18.51)	2.57 (1.15)	5.94 (2.3)
Eye Disorders	Subretinal Fluid	3	101.37 (32.49, 316.21)	101.14 (32.45, 315.23)	6.65 (5.22)	100.3 (38.71)

#### Documented adverse event signals

This study matched the adverse events recorded in the Selumetinib package insert with the adverse event signals identified in this research, revealing that most adverse events were included in our study and aligned with the common adverse reactions reported in the literature, such as those affecting the digestive system, skin, and visual system. Considering these adverse events are mainly related to the pharmacological action of Selumetinib, it is essential to maintain a high level of safety awareness during medication use to reduce the occurrence of adverse drug reactions.

In the ranking of adverse event signal strength, although there were only 4 reports of serous retinopathy, its signal strength ranked first among the adverse event signals, suggesting the strongest association with Selumetinib. However, current research on this adverse reaction is limited, and it cannot be confirmed whether it is caused by Selumetinib usage. When such adverse reactions occur, they should be reported promptly, and patients’ physical conditions should be closely monitored, with active intervention measures or discontinuation of the drug being considered.

#### New suspected adverse reactions

According to the disproportionality analysis, there were 20 positive PT signals that were not documented in the Selumetinib package insert. Excluding reports related to Selumetinib indications, three of the top 20 most frequently reported PTs not documented in the package insert included retinal detachment, peripheral edema, and oral mucositis. Additionally, among the top 20 PTs with the highest signal strength, six were not documented, including subretinal fluid, streptococcal pharyngitis, personality change, menstrual irregularities, increased intracranial pressure, and hydrocephalus. This study also identified new SOCs not listed in the package insert, such as rhabdomyolysis, serous retinopathy, urinary frequency, poor wound healing, ingrown nails, and proteinuria. Although these adverse reactions are not included in the Selumetinib package insert, they have occurred in real-world settings and show a strong correlation with Selumetinib. Clinicians should consider the risk of these adverse reactions when prescribing Selumetinib.

#### AE onset times

**FIGURE 4:**
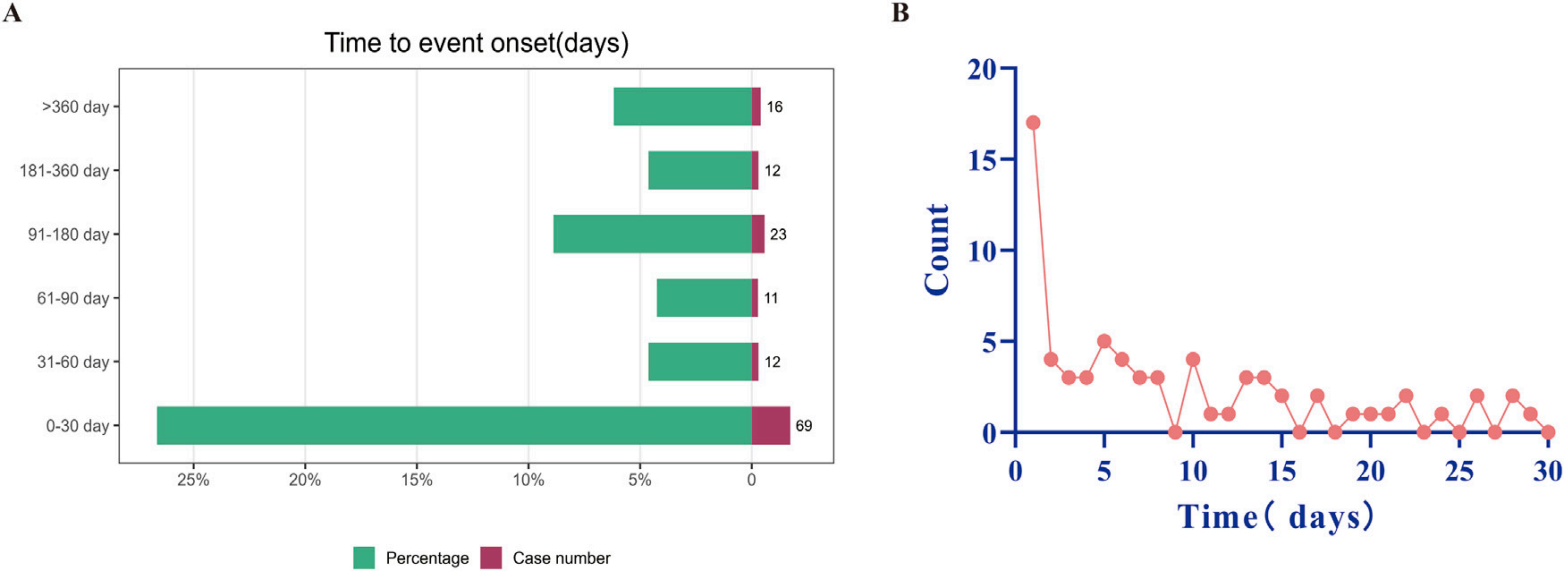
Timing of reported AEs. **(A)** Timing of reported AEs by month. **(B)** Timing of reported AEs by days of Selumetinib use within the first month. AE as adverse event.

Adverse events (AEs) associated with Selumetinib use were extracted from the database. After excluding patients with incomplete or inaccurate onset time information in FAERS, 143 AEs (29.42%) were reported, with a median onset time of 38 days. Approximately 48.25% of cases (n = 69) experienced AEs within the first month of starting Selumetinib (Figure 4A). The proportion of cases reporting AEs decreased significantly after 2 months (n = 12, 8.39%) and 3 months (n = 11, 7.69%). However, the proportion increased again between 3 and 6 months (n = 23, 16.08%). Notably, the highest number of AEs occurred on the first day of starting Selumetinib within the first month (n = 17, 24.63%) (Figure 4B).

## Discussion

NF1 is a rare disease that has seen no significant breakthroughs in drug development. Selumetinib, the first treatment for NF1, has instilled hope in managing the condition. Research on Selumetinib mainly focuses on its mechanism of action and clinical trials, with limited studies on recent real-world findings. To address this gap, we conducted a retrospective post-market pharmacovigilance study using the FAERS dataset. Our study aimed to comprehensively evaluate Selumetinib’s safety profile and provide insights into its real-world application.

Compared to females (37.04%), males reported more adverse reactions after using Selumetinib (42.59%), possibly because Selumetinib is mainly used to treat symptomatic, inoperable NF1-related plexiform neurofibromas (PNs) in patients aged 2–18, a condition more common in males than females. Clinicians should be vigilant about adverse reactions associated with Selumetinib as its clinical use is increasing. Our findings reveal that the reporting rate of adverse reactions linked to Selumetinib, resulting in all possible life-threatening conditions and death, is 12.96%. Hence, early detection of adverse reactions related to Selumetinib and prevention of severe adverse reactions are crucial for its clinical utility.

In the FAERS database, 48 PTs from 15 organ systems were identified as having disproportionate signals. Among all AE reports related to Selumetinib, the most relatively frequent include elevated blood creatine phosphokinase, rash, acneiform dermatitis, paronychia, and others, consistent with Selumetinib product labeling and previous findings. However, our results also highlight the importance of monitoring serious complications associated with Selumetinib use, such as rhabdomyolysis (Azizi et al., 2024). This discrepancy may be due to the small sample size and short follow-up duration in previous studies (Hummel et al., 2024).

Our study based on the FAERS database suggests that skin and subcutaneous tissue disorders are the most common side effects of Selumetinib (Peacock et al., 2024). A review by Paola Borgia et al. of a prospective study involving 20 patients treated with Selumetinib for NF1 yielded results highly consistent with our study (Borgia et al., 2024), namely that skin side effects are common, including xerosis, paronychia, and acneiform rash (Palmeiro et al., 2023). Therefore, because skin toxicity can affect quality of life and treatment compliance, we emphasize that addressing skin reactions is crucial for long-term management of patients receiving Selumetinib.

Drug-induced cardiotoxicity has always been a focus of concern for doctors, with common cardiotoxic drugs such as anthracyclines, trastuzumab, and immune checkpoint inhibitors. Based on the FAERS database, we conducted a retrospective analysis and found that all-level cardiotoxicity related to Selumetinib accounted for 4.88% of all AE reports, including decreased ejection fraction (n = 13), pericardial effusion (n = 4), and cardiac toxicity (n = 4). Therefore, combining this study with previous meta-analyses suggests that myocardial enzyme and echocardiography examinations should be performed before starting Selumetinib treatment (Han et al., 2024), every 3 months in the first year, every 6 months thereafter, and when clinically indicated (Caiffa et al., 2023).

Rhabdomyolysis syndrome refers to the destruction of the integrity of skeletal muscle cell membranes and the leakage of cell contents, including enzymes such as myoglobin and creatine phosphokinase (CPK), as well as toxic ions and small molecules into the blood, leading to a group of clinical syndromes often accompanied by life-threatening acute kidney injury when muscles are damaged by various factors. A retrospective study by Mary Kate Anderson showed that 76% of patients using selumetinib would experience an increase in CPK, and 9% of patients had an increase of ≥ grade 3. In addition, 7% of patients had their dose reduced due to CPK elevation, and one patient discontinued treatment due to muscle pain. Our study identified six cases of Selumetinib-related rhabdomyolysis. Given the kidney-damaging nature of rhabdomyolysis, this should be of great concern, and the causal relationship between the two should be clearly understood. Our study results suggest that CPK levels should be measured regularly before starting Selumetinib treatment, during treatment, and when clinically indicated. If this occurs, evaluate whether the patient has rhabdomyolysis or other causes of CPK elevation (Han et al., 2024).

In summary, previous clinical trials, such as the phase II study published in The New England Journal of Medicine (2016), reported that the most common AEs were mild to moderate skin reactions, gastrointestinal symptoms, and elevated CPK levels (Dombi et al., 2016). Our study corroborates these findings, highlighting skin and subcutaneous tissue disorders as the most frequently reported AEs in real-world settings. However, we also identified additional serious AEs, such as rhabdomyolysis, which were not documented in earlier trials, likely due to smaller sample sizes and shorter follow-up periods in controlled studies. Divergence from Controlled Studies: The identification of new AEs, such as rhabdomyolysis, underscores the limitations of clinical trials in capturing rare or long-term safety concerns. The spontaneous nature of FAERS data allows for the detection of AEs across a broader and more diverse population, providing insights that complement controlled studies. Alignment with Other Post-Market Studies: Recent pharmacovigilance studies on MEK inhibitors have similarly reported muscle-related AEs (Masuzawa et al., 2022). For instance, a 2024 review by Paola Borgia et al. identified skin toxicities and elevated CPK as significant concerns in pediatric patients treated with Selumetinib, findings consistent with our study (Borgia et al., 2024; Sadighi et al., 2024). These parallels reinforce the robustness of our signal detection methods and highlight shared challenges in the safety management of MEK inhibitors. These findings emphasize the need for targeted monitoring strategies, including early regular CPK testing, particularly during the initial weeks of treatment (Needle et al., 2024).

Despite the comprehensive nature of this study, several limitations must be acknowledged, along with potential strategies to mitigate their impact: 1. Underreporting Bias: The FAERS database relies on spontaneous reporting, which is inherently subject to underreporting. Clinically significant AEs might be underrepresented, particularly those with subtle or delayed onset. To address this, future studies should incorporate data from multiple pharmacovigilance databases, such as EudraVigilance or WHO Vigibase, to cross-validate findings and enhance signal detection. 2. Confounding Factors: The absence of demographic and clinical details, such as comorbidities and concurrent medication use, limits the ability to control for confounding variables. This may result in over- or underestimation of AE signals. Conducting case-control studies or nested cohort analyses with linked electronic health records can help establish causal relationships. 3 Lack of Causality Assessment: As the FAERS database does not require proof of causality, some reported AEs may not be directly attributable to Selumetinib. Employing methodologies like the Naranjo Adverse Drug Reaction Probability Scale in future investigations could provide a more robust causality assessment. 4. Small Sample Size: The rarity of NF1 and the recent approval of Selumetinib contribute to a limited sample size in the FAERS database, which may restrict the generalizability of results. Expanding the study as more data become available and pooling global post-market surveillance data can improve statistical power. 5 Selection Bias: The voluntary nature of reporting introduces selection bias, with reports often reflecting severe or unusual cases rather than mild or common AEs. This could skew the identified AE signals. Future studies should include prospective pharmacovigilance methods to capture a more representative sample of Selumetinib-treated patients. To strengthen the reliability of findings, future research could validate signals using complementary methods, such as *in vitro* experiments, animal models, or longitudinal clinical studies. By acknowledging these limitations and implementing the suggested strategies, subsequent research can build on these findings to provide a more nuanced understanding of Selumetinib’s safety profile. Collaborative efforts between regulatory agencies, clinicians, and researchers are essential to improve pharmacovigilance practices for rare diseases such as NF1.

## Data Availability

The raw data supporting the conclusions of this article will be made available by the authors, without undue reservation.
